# Evaluating the potential for evolutionary mismatch in Batesian mimics: A case study in the endangered smooth snake (*Coronella austriaca*)

**DOI:** 10.1111/eva.12679

**Published:** 2018-08-11

**Authors:** Janne K. Valkonen, Anni Mäkelä, Johanna Mappes, Andrés López‐Sepulcre

**Affiliations:** ^1^ Department of Biology and Environmental Science Centre of Excellence in Biological Interactions University of Jyväskylä Jyväskylä Finland; ^2^ CNRS UMR 7618 Institute of Ecology and Environmental Sciences Paris (iEES) Université Pierre et Marie Curie Jyväskylä Finland

**Keywords:** aposematism, behaviour, conflict, predation, reptiles

## Abstract

Many harmless organisms gain a survival advantage by mimicking venomous species. This is the case of the endangered smooth snake (*Coronella austriaca*), which mimics venomous vipers. Although this may protect the smooth snake against most of its natural predators, it may render them at greater risk of mortality from humans, who are more inclined to kill species, such as vipers, that they consider dangerous. This may cause an evolutionary mismatch, whereby humans may counteract the natural advantage of mimicry. We explore this possibility of evaluating the willingness of humans to kill smooth snakes versus the adder (*Vipera berus*), as well as their ability to discern them in the Åland Islands. Our results show that, even when respondents did not wish to kill the smooth snakes, these were often mistaken for adders, which they were willing to kill. Altogether, viper mimicry brought about a 2.3‐fold increase in the likelihood of smooth snakes being killed upon human encounter. These results open up the possibility that naturally selected mimicry can pose a threat to endangered snakes in human‐influenced habitats. We discuss the potential for this to be the case, and highlight the importance of protecting entire mimicry complexes, rather than single species, when the endangered species is a mimic.

## 1 INTRODUCTION

Evolutionary mismatch occurs when a previously adaptive trait becomes maladaptive in human‐altered environments. For example, sea turtle hatchlings, which are attracted to the brightest light (normally the sea), are nowadays instead directed towards streets by artificial lights (Witherington & Bjorndal, 1991); mayflies lay their eggs on tarmac because they have evolved to use polarized light as a cue for a water body (Kriska, Horváth, & Andrikovics, 1998).

In many societies, humans kill species that they consider dangerous such as venomous snakes and large carnivores, and this represents an important threat to those populations (e.g., Whitaker & Shine, 2000; Treves & Karanth, 2003; Akani, Eyo, Odegbune, Eniang, & Luiselli, 2013). However, natural selection has driven many harmless species to resemble dangerous ones to capitalize on their warning signals and avoid predators. This phenomenon, known as Batesian mimicry (Bates, 1862), can therefore potentially become maladaptive in human‐dominated environments if, rather than protecting from attack, resemblance to dangerous species increases the probability of attack (Valkonen & Mappes, 2014). Venomous snakes probably rank among the most feared animals, and intentional killings are common (Akani et al., 2013; Dodd, 1987; Greene, 1997). Many harmless snake species exhibit Batesian mimicry (Brodie & Brodie, 2004). A classical example is the nonvenomous king snake (genus *Lampropeltis*) mimicking the deadly coral snake (genus *Micrurus*) (Wallace, 1867). This brings into question whether humans pose a serious threat for harmless snake species whose natural antipredator strategy is to resemble a venomous species (Valkonen & Mappes, 2014).

**Figure 1:**
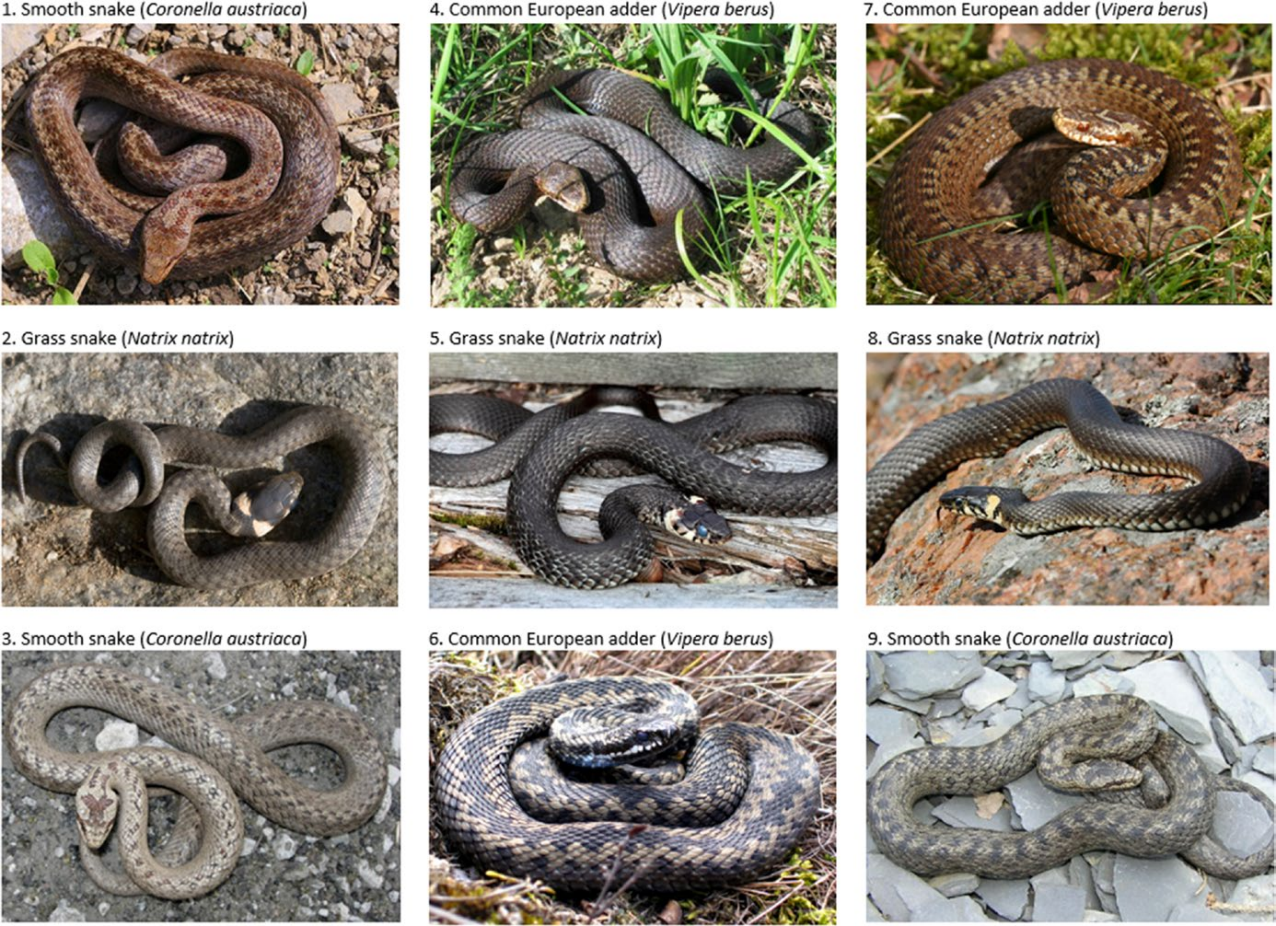
Pictures of smooth snakes (*Coronella austriaca*), adders (*Vipera berus*) and grass snakes (*Natrix natrix*) used for recognition task during the interviews

In this article, we investigate the potential for evolutionary mismatch of Batesian mimicry in an endangered population (Galarza, Mappes, & Valkonen, 2015) of smooth snake (*Coronella austriaca* Laurenti) in the Åland Islands (Finland). Smooth snakes exhibit a superficial resemblance of zigzag‐patterned European vipers (genus *Vipera*), including the adder (*Vipera berus* Linnaeus). The zigzag pattern of European vipers warns their predator about the venomousness of the snake (Niskanen & Mappes, 2005; Valkonen, Niskanen, Björklund, & Mappes, 2011b; Valkonen et al., 2012; Wüster et al., 2004), but it also reduces their detectability (Santos et al., 2014, 2017) and possibly hinders their capturing success by natural predators during attack (Forsman, 1995; Shine & Madsen, 1994). When threatened, smooth snakes also flatten their head to resemble the triangular shape of a viper's head and increase their mimicry (Figure 1) (Arnold, Burton, & Ovenden, 1978; Valkonen & Mappes, 2014; Valkonen, Nokelainen, & Mappes, 2011a). The smooth snake may therefore be easily misidentified as a viper by humans and killed. In Finland, the adder is the only venomous snake species and thus an important model species for mimics. However, adders are not protected by law in Finland, and it is legal to kill them, which may indirectly increase the threat to the similar yet endangered smooth snake.

When estimating the costs of mimicry, it is important to consider that other species that do not resemble adders may also be indiscriminately killed due to a general dislike of snakes. Mimicry would not incur an extra cost if humans are willing to kill any snake regardless of their degree of resemblance to venomous species. In this study, we illustrate how to do this using results from a questionnaire‐based study evaluating the attitudes of Åland Islanders towards adders, smooth snakes and a nonmimic snake, the grass snake (*Natrix natrix* Linnaeus), as well as their identification skills. With this, we show how to calculate the increased risk of smooth snakes being killed upon human encounter due to their viper mimicry.

## 2 METHODS

### 2.1 Study species and interview data

This study is based in the Åland Islands that are located in the Baltic Sea between Finland and Sweden. All three species included—the smooth snake (*Coronella austriaca*), the common adder (*Vipera berus*) and the grass snake (*Natrix natrix*)—are the only three snake species in the area. They are widely distributed across the Europe, and their ranges are largely overlapping (Arnold et al., 1978; Kreiner, 2007).

To evaluate people's attitude towards snakes and estimate the costs of viper mimicry upon human encounter, we interviewed and evaluated species recognition skills of 102 people in Åland throughout June 2014. To avoid possible confounding effects of unnatural behaviour in captivity and to provide interviewees the time they needed for species recognition, we chose to show photographs of snakes in their natural habitat instead of displaying captured live animals. We gathered interviews in supermarkets and malls from the municipalities of Mariehamn, Jomala, Hammarland, Sund and Lemland (approximately twenty people from each). Interviewees were first asked: 1) whether they had seen snakes during the last year and 2) whether they had seen them in their yards, nature or both. Afterwards, they were asked to 3) identify pictures of all three snake species in Åland: smooth snake, adder and grass snake (Figure 1). We did this by showing three pictures of each snake species (total of nine pictures) to each interviewee. We randomized the order of the pictures, yet kept the same orders for all interviews. The interviewees answered what species they believed the picture to be or declared not to recognize the species. We recorded answers of interviewees as correct, incorrect or unknown. We then asked: 4) whether they were willing to kill snakes. At last, we noted if they declared a disposition to kill snakes, they were asked 5) whether they would kill only venomous adders or any snake species encountered.

### 2.2 Data analyses

To measure the efficacy of viper mimicry to a human eye, we tested how often humans think a smooth snake is an adder compared to nonmimetic grass snakes (to control for a generic perception of all snakes as adders) and how many times they think an adder is an adder (to account for the general level of identification error). To do so, we modelled the identification tests as a generalized linear mixed model (GLMM) with a binomial error governing the probability of an image being identified as an adder. We included the correct species as a fixed explanatory factor parameterized such that adders represent the intercept. To reflect the sampling structure (every individual was given the same set of pictures to identify), we included picture and interviewee identity as random effects. This accounts for the fact that some pictures may be easier to identify than others, and some individuals may be generally better than others identifying snakes.

Human misidentification of smooth snakes will only create a mimicry‐related cost if humans are willing to kill selectively (i.e., only want to kill the venomous adder), rather than to kill all snakes indiscriminately. If humans regard all snakes as equally dangerous, mimic snakes would not be expected to incur any further cost than nonmimics. Let us consider the baseline probability that an encountered human is willing to kill a snake regardless of species to be μ_0_. This is a cost expected to be equal for both the smooth snake and the grass snake. On the other hand, any given species of snake will also incur a further cost which is composed of 1) the probability that the encountered human only wishes to kill venomous snakes (adders) μ_v_, times, 2) the probability of that human mistakenly identifying that snake as a venomous adder ε_i_ where *i* is species‐specific. While this should apply equally to the smooth and the grass snake, it is expected that it will be much higher in smooth snakes due to their resemblance to vipers. We can therefore conceptualize the increase in cost due to viper resemblance as a risk ratio ϕ_*i*_ between the total risk of a harmless snake being killed upon human encounter (including being mistaken for a viper, $\mu_{0}+\mu_{v}\varepsilon_{i}$) and the risk attributable merely to the fact of being a snake $\mu_{0}$:

(1) $$\emptyset_{i}=\frac{\mu_{0}+\mu_{v}\varepsilon_{i}}{\mu_{0}}$$

We estimated μ_0_ as the proportion of individuals interviewed that were willing to kill all snakes found and calculated the standard error using a simple binomial generalized linear model with a constant intercept and a logit link. The proportion of individuals who were willing to kill only venomous snakes (adders) was used in the same way to estimate μ_v_.

Because there are potential differences in the identification skills of people with different attitudes towards snakes (e.g., one might expect that people willing to kill selectively only venomous snakes are better at identifying them that people who are willing to kill indiscriminately), we calculated ε_i_ by rerunning the identification GLMM described above only on data for individuals that are willing to kill venomous snakes selectively. We also more explicitly tested whether identification skills were related to attitude using a GLMM on the identification tests with the probability of making a mistake as a binomial response variable (with logit link), attitude (do not kill, kill indiscriminately and kill selectively) as an explanatory fixed factor and random effects for picture and interviewee ID.

To calculate the error in the estimation of ϕ_*i*_, we performed Monte Carlo simulations where we drew 10,000 random samples from each parameter estimate distributions and repeated the calculation in Equation 1 to extract a distribution of values for ϕ_*i*_. For comparison, we calculated ϕ_*i*_ for both the smooth snake and the grass snake, where we expect the misidentification component to be much lower. All statistical analyses were conducted in R 3.3.1, using function glmer in package lme4 for the GLMM (Bates, Maechler, Bolker, & Walker, 2015).

## 3 results

**Figure 2:**
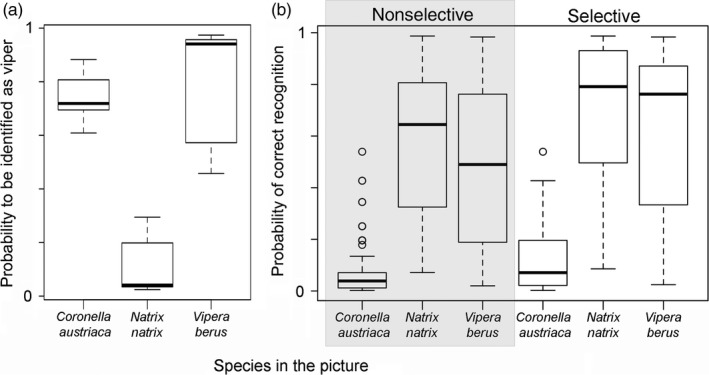
Estimated recognition probabilities of smooth snakes (*Coronella austriaca*), adders (*Vipera berus*) and grass snakes (*Natrix natrix*). Smooth snakes were mistaken as vipers nearly as often as vipers where recognized, whereas grass snakes were significantly less likely mistaken (a). Participants who were willing to kill selectively only vipers had slightly better recognition skills than people who were willing to nonselectively kill all snakes or not willing to kill snakes at all (b)

The majority (94%) of respondents saw at least one snake in the past year, and most of them (69.6%) declared not to be willing to kill snakes indiscriminately. Only 9.8% of interviewees declared to be willing to kill snakes regardless of the species, and 20.6% would only kill vipers. In the latter group, the probability of confusing a smooth snake for a viper was of 67.7%, while they would only confuse grass snakes for vipers 7.2% of the time. While grass snakes in pictures were significantly less likely to be identified as viper than vipers themselves, smooth snakes were identified as vipers just as often as vipers (Table 1, Figure 2a). This supports our hypothesis that humans confuse smooth snakes for vipers. Moreover, the probability of misidentifying a snake in the picture was lower for people who were willing to kill only venomous snakes compared to people who were either willing to kill indiscriminately or not kill any (Table 1, Figure 2b).

**Table 1:** Binomial GLMM on the probability of identifying a snake as a viper as a function of true species identity

Random effects	Variance			
Picture ID	0.134			
Interviewee ID	1.128			

Fixed effects	Estimate	*SE*	*Z*	*p*‐value
(Intercept) *Vipera berus*	2.114	0.679	3.114	0.002
*Coronella austriaca*	−0.983	0.928	−1.060	0.289
*Natrix natrix*	−4.841	0.979	−4.941	<0.001

Given the above results, the parameterization of Equation 1 yielded a risk ratio of 2.3 (CI_95%_ = 1.65–3.78), meaning that smooth snakes more than doubled their probability of being killed upon human encounter due to their resemblance to vipers.

## 4 DISCUSSION

We have shown how to calculate the increased cost of mimicry induced by humans upon encounter. In reality, whether this cost translates into an evolutionary mismatch, and therefore selection against mimicry, will depend on the relative encounter rates of mimics with human vs. natural enemies.

Snakes are often killed because humans regard them as a threat (Akani et al., 2013; Dodd, 1987; Greene, 1997). Although adder bites are a minor threat to humans in Finland (Grönlund, Vuori, & Nieminen, 2003), adders are still often abhorred and killed. Our survey shows that 30.4% of human respondents had a negative attitude against snakes (i.e., they were willing to kill snakes at least in some circumstances). In accordance with the hypothesis of Valkonen and Mappes (2014), our results show that viper mimicry in smooth snakes increases their risk of being killed by humans who dislike adders. We show that smooth snakes are often misidentified as adders, which more than doubles their risk of being killed by humans. This can challenge, and potentially select against, the smooth snake's natural antipredator strategy of mimicking venomous vipers (Valkonen & Mappes, 2014).

By bad luck, our study does not allow us to estimate neither total human‐induced mortality nor the natural benefits of viper mimicry for the smooth snake. However, a previous field experiment tested the advantage of bearing a viper‐like phenotype against birds of prey and found that it reduced overall predation risk by a factor of 3 (CI_95%_ = 2.44–3.88) (Valkonen et al., 2012). This benefit of viper mimicry is of similar magnitude to the 2.3‐fold (CI_95%_ = 1.65–3.78) increase in human‐induced mortality. Although these estimates are relative risks and do not reflect absolute values of mortality, they suggest that viper mimicry has the potential to become detrimental in heavily human‐inhabited environments.

Our results show that humans mistake smooth snakes for adders with very high probability. It is evident that the accuracy of mimicry (up to a certain level) increases the probability that a predator mistakes harmless mimics for dangerous models and thus refrains from attack (e.g. Rowland, Ihalainen, Lindström, Mappes, & Speed, 2007; Sherratt, 2002). It can therefore be expected that better copies of venomous model species were better protected from natural predation and selected for by natural selection. However, this may not hold against specialist predators that are not paying a cost for attacking the venomous model species and possibly favouring them in their diet (Endler & Mappes, 2004; Valkonen et al., 2012). In the same way, as long as it is not costly for humans to attack vipers, they will likely continue to do so.

Species identification errors can limit the effectiveness of conservation policy when it relies on the general public's species recognition ability. For example, in Australia, people are encouraged by many politicians to kill invasive cane toads (*Rhinella marina*) (Somaweera, Somaweera, & Shine, 2010 *)*, yet this can cause a severe threat to native frog species due to the difficulties of identification (discussed in Somaweera et al., 2010 and references therein). In the same way, protected species resembling actively harvested game species can suffer increased mortality caused by hunter misidentification. For example, protected crested coots (*Fulicula cristata*) benefit from group living in mixed flocks with the similar‐looking common coot (*Fulicula atra*) through predator protection or group foraging. However, common coots are a common game species, which leads to a high risk of accidental killings of the endangered crested coot during game season (Martínez‐Abraín, Viedma, Bartolomé, Gómez, & Oro, 2007).

These results highlight a potentially important conservation problem in mimicry systems, where endangered species are dependent on the abundance of its human‐abhorred model species. This is different from the cases where, for example, hunters are misidentifying and shooting protected species. That is because the mimic is reliant on the abundance of its model(s), which is likely not the case between game species and their protected relatives and bycatch. We are not aware of other studies showing a mimicry system where an endangered mimic relies on resemblance of a human‐abhorred model, decreasing the adaptive value of their mimicry through human‐induced mortality. However, we do not expect such cases to be uncommon, given the number and variation of endangered species.

Human killing of species in mimicry systems can also have indirect effects on the population dynamics of prey communities. That is because mimicry as a predatory defence is dependent on the abundance of co‐mimics and models (Honma, Takakura, & Nishida, 2008; Huheey, 1964; Joron & Mallet, 1998; Lindström, Alatalo, & Mappes, 1997; Mallet, 1999). In Batesian mimicry—were edible prey are mimicking toxic models (Bates, 1862)—predator avoidance learning is reinforced by unpleasant encounters with the defended model, whereas it is degraded by each rewarding encounter with the edible mimic. Because of this, the protection of mimicry against natural predators decreases if the abundance of models decreases (Huheey, 1964; Lindström et al., 1997).

One can think of other types of mimicry where it may be important to consider the protection of whole mimicry complexes. In Müllerian mimicry, two or more defended species share their appearance and the costs of predator avoidance learning (Müller, 1879). As predator avoidance learning is dependent on the number of unrewarding encounters with defended prey, the most abundant species is expected to pay most of the cost of predator education (Honma et al., 2008; Joron & Mallet, 1998; Mallet, 1999). Even if the most abundant species is not of conservation concern, a reduction in its population due to human killings will increase the cost of predation on a less abundant mimic, because of the lower availability of interspecific models to train naïve predators.

Considering mimicry could, for example, be important in the conservation of pollinator bees. Pollinator bees have both Batesian (hoverflies) and Müllerian (wasps) mimics that share with each other a black and yellow coloration. A reduction in wasp abundance would reduce bee protection from naïve predators. This increase in predation risk could be further accentuated by the increase in the relative numbers of harmless hoverflies in the community, thus possibly leading to the loss of efficacy of the mimicry as a natural antipredator strategy.

Theoretical expectations of frequency dependence, as well as disturbances in species frequencies in mimic‐model complexes, have been well studied (see e.g., Honma et al., 2008; Huheey, 1964; Joron & Mallet, 1998; Lindström et al., 1997; Mallet, 1999). However, the direct and indirect human‐induced fitness costs for the mimetic species that resemble an abhorred model species warrant further study to reveal the extent to which it creates an evolutionary mismatch. We hope that the example presented here encourages further studies on similar systems across taxa. Protecting only endangered mimics may not be a sufficient conservation strategy. Rather, it might be necessary to protect entire mimic‐model complexes, through educational and legal initiatives, in order to discourage humans from killing any of the involved species and avoid the loss of endangered individuals to misidentification.

## CONFLICT OF INTEREST

None declared.

## DATA ARCHIVING STATEMENT

Data available from the Dryad Digital Repository: https://doi.org/10.5061/dryad.vv34k10.
